# Prognostic Significance of *O*-GlcNAc and PKM2 in Hormone Receptor-Positive and HER2-Nonenriched Breast Cancer

**DOI:** 10.3390/diagnostics11081460

**Published:** 2021-08-12

**Authors:** Wen-Ling Kuo, Lin-Lu Tseng, Che-Chang Chang, Chih-Jung Chen, Mei-Ling Cheng, Hsin-Hung Cheng, Meng-Jen Wu, Yu-Lun Chen, Ruei-Ting Chang, Hsiang-Yu Tang, Yong-Chen Hsu, Wen-Jye Lin, Cheng-Yuan Kao, Wen-Ping Hsieh, Hsing-Jien Kung, Wen-Ching Wang

**Affiliations:** 1 Division of Breast Surgery, General Surgery, Department of Surgery, Chang Gung Memorial Hospital Linkou Medical Center, Taoyuan City 33305, Taiwan; sylvie5285@gmail.com; 2 Institute of Molecular and Cellular Biology and Department of Life Sciences, National Tsing-Hua University, Hsinchu City 30013, Taiwan; linlutseng@gapp.nthu.edu.tw (L.-L.T.); fabiencheng@gmail.com (H.-H.C.); mengjhen@gmail.com (M.-J.W.); utopia_peter@hotmail.com.tw (Y.-L.C.); 3 The Ph.D. Program for Translational Medicine, College of Medical Science and Technology, Taipei Medical University, Taipei City 11031, Taiwan; ccchang168@tmu.edu.tw (C.-C.C.); bulesky7879@hotmail.com (R.-T.C.); 4 Department of Pathology and Laboratory Medicine, Taichung Veterans General Hospital, Taichung City 40705, Taiwan; cjchen1016@gmail.com (C.-J.C.); shuyongjen@hotmail.com (Y.-C.H.); 5 School of Medicine, Chung Shan Medical University, Taichung City 40201, Taiwan; 6 Department of Biomedical Sciences, College of Medicine, Chang Gung University, Taoyuan City 33302, Taiwan; chengm@gap.cgu.edu.tw; 7 Metabolomics Core Laboratory, Healthy Aging Research Center, Chang Gung University, Taoyuan City 33302, Taiwan; tangshyu@gmail.com; 8 Immunology Research Center, National Health Research Institutes, Miaoli County 35053, Taiwan; wjlin@nhri.edu.tw (W.-J.L.); chengyuankao@nhri.edu.tw (C.-Y.K.); 9 Institute of Statistics, National Tsing Hua University, Hsinchu City 30013, Taiwan; wphsieh@stat.nthu.edu.tw; 10 Graduate Institute of Cancer Biology and Drug Discovery, Taipei Medical University, Taipei City 11031, Taiwan; hkung@tmu.edu.tw; 11 Department of Biochemistry and Molecular Medicine, University of California Davis School of Medicine, University of California Davis Cancer Centre, Sacramento, CA 95817, USA

**Keywords:** *O*-GlcNAc, PKM2, HR^+^/HER2^−^ luminal breast cancer, metabolism, prognosis

## Abstract

Predictive metabolic biomarkers for the recurrent luminal breast cancer (BC) with hormone receptor (HR)-positive and human epidermal growth factor receptor type 2 (HER2)-negative are lacking. High levels of *O*-GlcNAcylation (*O*-GlcNAc) and pyruvate kinase isoenzyme M2 (PKM2) are associated with malignancy in BC; however, the association with the recurrence risk remains unclear. We first conduct survival analysis by using the METABRIC dataset to assess the correlation of PKM2 expression with BC clinical outcomes. Next, patients with HR^+^/HER2- luminal BC were recruited for PKM2/*O*-GlcNAc testing. Logistic regression and receiver operating characteristic curve analysis were performed to evaluate the 10-year DFS predicted outcome. Survival analysis of the METABRIC dataset revealed that high expression of PKM2 was significantly associated with worse overall survival in luminal BC. The high expression of *O*-GlcNAc or PKM2 was a significant independent marker for poor 10-year DFS using immunohistochemical analysis. The PKM2 or *O*-GlcNAc status was a significant predictor of DFS, with the combination of PKM2–*O*-GlcNAc status and T stage greatly enhancing the predictive outcome potential. In summary, *O*-GlcNAc, PKM2, and T stage serve as good prognostic discriminators in HR^+^/HER2^−^ luminal BC.

## 1. Introduction

The incidence of breast cancer (BC), a common malignancy in women, is rising sharply in East Asia, particularly in relatively young or premenopausal women, possibly due to a Westernized lifestyle and environmental factors [1]. BC is a highly heterogeneous disease and is molecularly classified into five intrinsic subgroups based on gene expression signatures, which was modified for clinical utility using pathologic parameters and receptor status by immunohistochemistry (IHC) in paraffin-embedded sections [2]: (1) luminal A (human epidermal growth factor receptor type 2 (HER2)^−^, estrogen receptor (ER) and/or progesterone receptor (PR)^+^, with Ki-67 < 20%), (2) luminal B (HER2^−^, ER, and/or PR^+^, with Ki-67 > 20% or grade (3)), (3) luminal HER2 (HER2^+^, ER, and/or PR^+^), (4) HER2 (HER2^+^, ER^−^, and PR^−^), and (5) triple-negative BC (TNBC; ER^−^, PR^−^, and HER2^−^).

The risk of relapse in the Luminal B subtype, which is characterized by positive hormonal receptors (HRs), non-amplified *HER2*, a higher tumor grade, a higher Ki-67 level, and more aggressive tumor behavior, is high and increases at a constant rate up to 10 years after diagnosis [3]. The incidence of the luminal subtype is also higher (i.e., 67%) among cases of premenopausal BC, 72% of which are luminal B with a high tumor grade (grade 2–3) and are associated with worse prognoses than that of luminal A [4,5,6]. Although luminal A and B harbor overlapping intrinsic markers and distinct clinical and molecular features, genomic analyses have revealed higher frequencies of DNA amplification and chromosomal alterations, as well as a higher tumor mutational burden in luminal B than in luminal A, which offers growth advantage independent of estrogen stimulation [7].

Clinicopathological parameters, such as tumor diameter and nodal status (TN) as well as tumor grade, are strongly associated with long-term disease-free survival (DFS) in HR^+^/HER2^−^ BC [8]. The Nottingham prognostic index (NPI) is the earliest tool combining nodal status, tumor size, and histological grade to predict outcomes in early BC [9,10]. The clinical treatment score post-5 years (CTS5) integrates tumor size, tumor grade, patient age, and the number of nodes (https://www.cts5-calculator.com (accessed on 7 April 2020)), and provides another useful web-based tool to estimate the risk of late recurrences [11,12]. As a continuous variable, the CTS5 score can be used to categorize patients into low, intermediate, and high-risk groups (<5%, 5–10%, and >10% distant recurrence risk, respectively) after 5 years of endocrine therapy for postmenopausal women. A recent validation study of a large sample of patients with BC using SEER (Surveillance, Epidemiology, and End Results) data has revealed a comparable prognostic power in both premenopausal and postmenopausal patients with BC [13]. In contrast, Sestak et al. recently evaluated CTS5 in the TAILORx trial and reported that the prognostic value is lower in patients aged 50 years or younger than in older patients [14].

Multigene expression signature is clinically useful to predict the relapse risk in HR^+^/HER2^−^ BCs [15]. For instance, OncotypeDx (a 21-gene assay) [16,17,18] and MammaPrint (a 70-gene assay) [19] offer recurrence scores to determine the benefit of adjuvant chemotherapy. EPclin score by EndoPredict [20], ROR score by Prosigna [21], and a two-gene ratio (*HOXB13*/*IL17BR*) by Breast Cancer Index [22] are also commercial risk-predicting panels. These gene-expression-based assays are primarily based on the analysis of marker genes, which may offer some clues for understanding complex mechanisms underlying BC progression.

An emerging approach for the prediction of recurrence and risk stratification involves metabolic panels, as cancer cell proliferation and progression are fueled by metabolic reprogramming [23,24,25,26]. Warburg metabolism, which diverts glycolytic flux into aerobic glycolysis, is observed in proliferating tumors [25]. In addition, increasing evidence suggests that upregulated *O*-GlcNAcylation of key factors contributes to cancer progression [27,28]. The sugar substrate UDP-*N*-acetylglucosamine (UDP-GlcNAc) generated from the hexosamine biosynthetic pathway (HBP) is utilized for the *O*-GlcNAc modification [27,28]. HBP integrates nutrient status from the metabolism of glucose, amino acids, and fatty acids and is involved in the regulation of signal transduction and metabolic reprogramming [29]. Notably, enhanced *O*-GlcNAcylation is important for BC malignancy, metastasis, and endocrine resistance [30,31,32,33].

PKM2, a glycolytic enzyme (conversion of phosphoenolpyruvate into pyruvate), serves as a control point in the metabolic network of cancer cells [34,35]. Metabolites (such as fructose-1,6-bisphosphate, serine, phenylalanine) and post-translational modifications regulate PKM2′s pyruvate kinase activity and hence the carbon flow to regulate the Warburg metabolism [34,35]. PKM2 exhibits non-metabolic functions, acting as a coactivator of HIF-1α, Stat3, and β-catenin pathways in the nucleus [36,37] and exhibiting protein kinase activity toward H3 and Stat3 [38,39,40]. PKM2 also partners with the oncogenic demethylase KDM8, which promotes the nuclear translocation of PKM2 and hence Warburg metabolism [41,42]. Notably, PKM2 is overexpressed in BC and is associated with poor clinical outcomes in a large-scale analysis [42] and in a meta-analysis of breast tumors [42,43]. Furthermore, an elevated degree of *O*-GlcNAcylated PKM2 is found in breast tumors [31]. *O*-GlcNAcylation of PKM2 at threonine 405 and serine 406 promotes tumor growth using a BC xenograft model [44]. Overexpression of PKM2 [45,46] and *O*-GlcNAcylation [30] have been indicated to confer a selection progression advantage to breast cancer. However, whether the expression of *O*-GlcNAc and/or PKM2 is associated with clinical outcomes in different BC subtypes remains unclear. In this study, we evaluated the clinical relevance of PKM2 expression in luminal tumors using the Metabric dataset. We also investigated the association between the expression of PKM2, *O*-GlcNAc, histological grade, and clinical outcomes in a retrospective cohort of endocrine-treated HR^+^/HER2^−^ patients by IHC and survival analysis, with a focus on the risk of recurrence.

## 2. Materials and Methods

### 2.1. Dataset Analysis

The METABRIC dataset [47] retrieved from (https://www.cbioportal.org (accessed on 25 March 2021)) was used to evaluate the correlation between PKM2 expression and clinical outcome in luminal tumors. The gene mRNA expression levels were divided into quartiles. A survival analysis was performed between the lowest group (bottom 25%) and the highest group (top 25%) using a log-rank test.

### 2.2. Study Subjects

**Figure 1:**
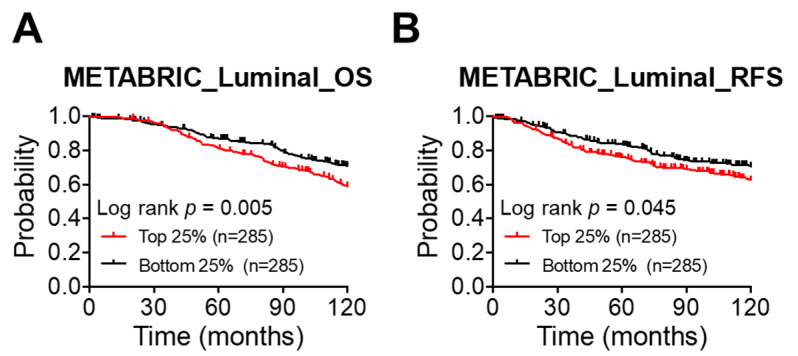
High expression of PKM2 is significantly associated with worse overall survival in luminal tumors. (**A**,**B**) A 10-year overall survival (**A**) and relapse-free survival (**B**) analysis based on the expression level of PKM2 in luminal tumors. Data were obtained from the METABRIC online dataset. The mRNA expression levels of PKM2 were divided into quartiles. An analysis between the top quartile group (top 25%) and the bottom one (bottom 25%) was then compared by a log-rank test.

A total of 3166 HR^+^/HER2^−^ luminal BC patients who had received therapy at Chang-Gung Memorial Hospital (CGMH), Linkou, Taiwan (2005–2013) were reviewed based on the pathological and clinical properties. The diagnostic criteria for HR^+^/HER2^−^ luminal BC was based on the receptor status (ER, PR, and HER2) by IHC and *HER2* amplification by fluorescence in situ hybridization (FISH) in invasive cancer as the following: HR^+^, more than 1% positive for ER or PR; HER2^−^, an IHC score of 0 to 1+, or 2+ with non-amplified *HER2* by FISH. One hundred and sixty-nine HR^+^/HER2^−^ patients who had concluding pathologic reports and no other malignancies were firstly selected. Seven patients were excluded for the following reasons: 4 cases with incomplete initial diagnostic information, 2 with mucinous carcinoma, and 1 with no available formalin-fixed paraffin-embedded block of the primary tumor. The final number of enrollments was 162, including 50 patients with documented recurrence and 112 patients without any recurrence over 10 years (Figure 1 and Table 1). Demographic factors of this retrospective cohort included age, history of diabetes, ER and PR status, tumor size, grade, TN stage, surgical treatment, chemotherapy use, endocrine therapy, and radiotherapy. The time and location of recurrence were documented. The study protocols have been approved by the Institutional Review Board of CGMH (IRB#201700716A3).

### 2.3. IHC Assessment

Consecutive paraffin-embedded serial sections of specimens (*n* = 162) were obtained from CGMH for single IHC staining of PKM2 and *O*-GlcNAc. The specimens were deparaffinized and rehydrated with xylene (Sigma-Aldrich) and ethanol (100% ethanol for 10 min, 95% ethanol for 5 min, 80% ethanol for 3 min, 70% ethanol for 3 min, 50% ethanol for 2 min, and then deionized water for 5 min). For antigen retrieval, specimens were incubated with citrate buffer at pH 6.0 (10 mM citrate, 0.05% Tween 20) and autoclaved at 121 °C for 20 min. The immunostaining procedures were performed using Novolink Polymer Detection System (Leica) according to the manufacturer’s manual. In brief, tissues were stained with anti-*O*-GlcNAc or anti-PKM2 for 60 min at room temperature. The working dilution of the primary antibody was 1:50 for anti-*O*-GlcNAc (838004, Biolegend, San Diego, CA, USA) and 1:100 for anti-PKM2 (D78A4, Cell Signaling Technology, Danvers, MA, USA). The IHC results were reviewed blindly by two pathologists (CJ Chen and YC Hsu) based on two parameters: the staining intensity score (0 = negative; 1 = weak; 2 = moderate; and 3 = strong), and the percentage of immunopositive cells (0–100) [48,49]. The Q score (0–300) was calculated by multiplying these two parameters. Two groups were stratified based on the Q score: low expression (Q ≤ mean) and high expression (Q > mean). Kaplan–Meier analysis was conducted to evaluate the clinical relevance.

### 2.4. Statistical Analysis

A Chi-square test was performed to compare clinicopathological properties of the patients with categorical variables. Kaplan–Meier analysis was conducted to assess overall patient survival, which was estimated as the time from diagnosis until death or until last follow-up. The log-rank test was used to determine the statistical significance between groups. Spearman’s correlation was performed to evaluate the status of *O*-GlcNAc, PKM2, or CTS5 with each of the clinical parameters. Differences were considered significant at *p* < 0.05. All *p*-values were obtained from 2-sided tests.

Logistic regression analysis was used to assess the contribution of the expression of *O*-GlcNAc and PKM2 and/or the CTS5 score in prognosis. A *p*-value of less than 0.05 was considered significant. The receiver operating characteristic (ROC) curve was plotted based on the set of sensitivity and specificity. The area under the curve (AUC) was computed using numerical integration of the ROC curves. Cox regression analysis was conducted to characterize the effect of one (*O*-GlcNAc, PKM2, invasive tumor size, T stage, or CTS5) or multiple variables (*O*-GlcNAc, PKM2, and/or a clinical parameter) upon the time of a DFS event takes to happen. Univariate and multivariate Cox regression, as well as logistic regression analyses, were performed using IBM SPSS, version 25 (IBM Corp. New York, NY, USA).

## 3. Results

### 3.1. Clinical Relevance of PKM2 Expression in Luminal Tumors

PKM2 is overexpressed in the breast tumors [42] and associated with poor BC prognosis in a meta-analysis [43]. We sought to further evaluate the clinical relevance of *PKM2* expression in the luminal tumors using the METABRIC cohort defined by PAM50 [47]. Figure 1A shows that patients with high PKM2 expression exhibited a significantly worse 10-year survival than did those with low PKM2 expression (top 25% vs. bottom 25%, *p* = 0.005). A significantly worse relapse-free survival was also found for the top-quartile group as compared with the bottom-quartile one (top 25% vs. bottom 25%, *p* = 0.045) (Figure 1B). We also evaluated the clinical relevance of *PKM2* expression in the basal and HER2 subtypes of BC tumors using the METABRIC cohort. Figure S1A shows that the basal subtype exhibited a significantly worse 10-year relapse-free-survival (RFS) (top 25% vs. bottom 25%, *p* = 0.046) but no significant difference in a 10-year overall survival (OS) outcome for the PKM2-high expression group. There was no significance for the HER2 subtype (Figure S1B).

These results suggested that high expression of PKM2 is associated with a worse clinical outcome in luminal tumors.

### 3.2. High PKM2 and High O-GlcNAc and Predicts Poor Prognosis in HR^+^/HER2^−^ BC

**Figure 2:**
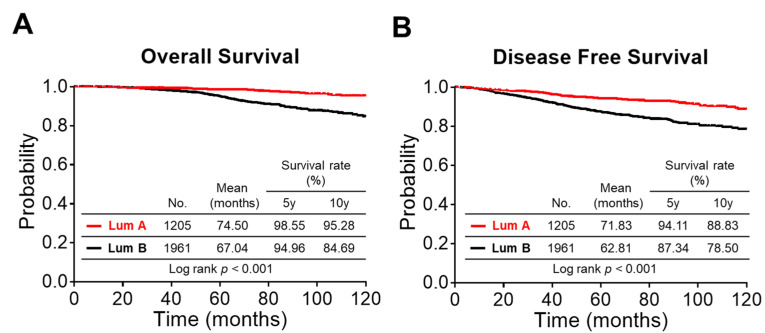
Survival analysis for HR^+^ luminal BC at Chang Gung Memorial Hospital (2005–2013). (**A**,**B**) Overall survival (**A**) and DFS (**B**) curves for HR^+^ luminal BC at Chang Gung Memorial Hospital (2005–2013). Lum A, luminal A (*n* = 1205); Lum B, luminal B (*n* = 1961). For group comparisons, *p*-values were determined by a log-rank test.

Patients with HR^+^/HER2^−^ luminal BC (*n* = 3166, 2005–2013 at Chang Gung Memorial Hospital) were classified into two groups based on pathological and IHC features: luminal A (ER and/or PR^+^, Ki67 ≤ 20%, or grade 1) and luminal B (ER and/or PR^+^, Ki67 > 20%, or grade 2 or 3). The luminal B group exhibited a significantly lower survival rate than that of the luminal A group (10-year overall survival (OS), *p* < 0.001; 10-year DFS, *p* < 0.001) (Figure 2A,B), revealing the substantial outcome difference between the two subtypes in our hospital-based population.

**Figure 3:**
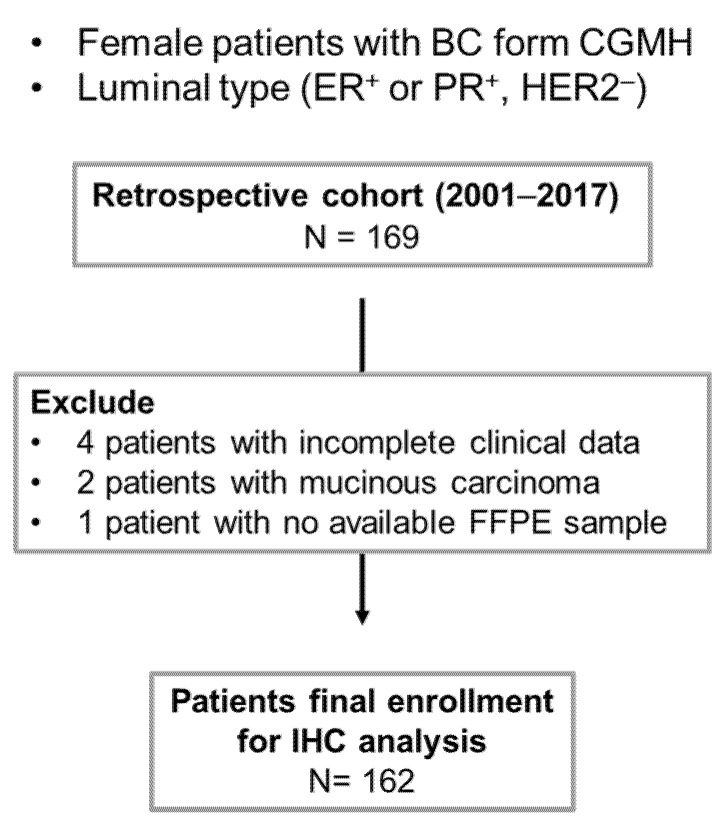
Flow diagram of patient selection in this study. One hundred and sixty-nine HR^+^/HER2^−^ patients who had pathologic reports and no other malignancies diagnosed in 2005–2013 were considered for this retrospective study. Seven were excluded because of incomplete clinical follow-up data (*n* = 4), mucinous carcinoma (*n* = 2), or no available formalin-fixed paraffin-embedded block of the primary tumor (*n* = 1).

We next evaluated the clinical relevance of PKM2 abundance in *HR^+^/HER2^−^* luminal BC using IHC analysis. Additionally, we evaluated the clinical association with respect to *O*-GlcNAc expression because of its implication in BC progression and endocrine resistance [30,31,32,33]. A retrospective cohort study was conducted on 162 subjects (non-recurrent, *n* = 112, median (interquartile range (IQR)) age, 51.0 (16.0) years; recurrent, *n* = 50, median (IQR) age, 53.0 (22.0) years) who had received BC therapy and were followed up until December, 2018 (median period, 123.4 months, IQR, 107.4–134.1 months) (Figure 3 and Table 1).

**Figure 4:**
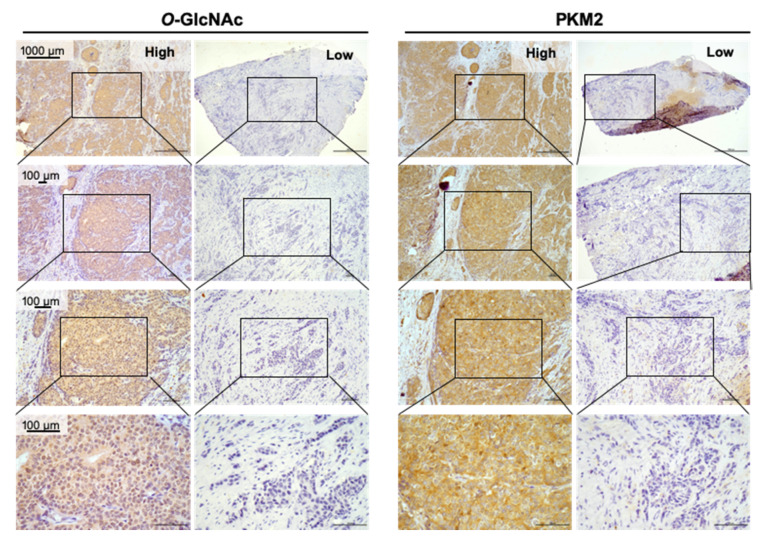
Representative images of IHC profiles. *O*-GlcNAc and PKM2 were immunostained for each of the BC specimens (*n* = 162). Low, Q ≤ mean; High, Q > mean. The images are enlarged in series (box, the enlarged portions).

*O*-GlcNAc and PKM2 were characterized by IHC staining for each of the samples (Figure 4). We first conducted Spearman’s correlation analyses of the status of *O*-GlcNAc, PKM2, or CTS5 with each of the clinical parameters. We were nonetheless not able to perform analysis for the Ki67 index because of 83 missing values. As shown in Table S1, the Spearman’s rank correlation coefficient of *O*-GlcNAc with each of the clinical parameters was low (correlation coefficient = −0.12–0.14), indicating low or no correlation. Similar results were also obtained for the Spearman’s rank correlation of PKM2 with each of the clinical parameters (Table S2; correlation coefficient = −0.293‒0.149). On the other hand, CTS5 includes the information of tumor size, tumor grade, and the number of nodes, exhibited a strong correlation with invasive tumor size, SBR Grade, T stage, N stage, or Stage (Table S3, correlation coefficient ≥0.679). These results together suggest that there was no or low correlation of PKM2 or *O*-GlcNAc expression with each of the clinical parameters in this retrospective cohort. Univariate Cox regression analysis revealed that invasive tumor size (*p* = 0.020), T stage (*p* = 0.004), *O*-GlcNAc expression (*p* < 0.001), or PKM2 expression (*p* = 0.048) was significantly associated with DFS whereas CTS5 (*p* = 0.109) had a non-significant impact (Table S4).

**Figure 5:**
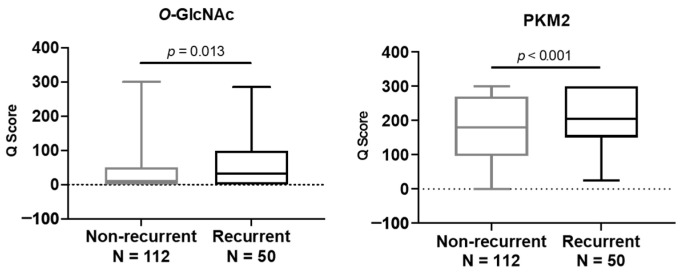
High *O*-GlcNAc or high PKM2 expression is significantly associated with the risk of recurrence. For group comparisons, *p*-values were determined by a two-tailed Student’s *t*-test.

To assess the expression level of *O*-GlcNAc or PKM2 in relation to recurrence risk, patients were stratified into a non-recurrent or a recurrent group. As shown in Figure 5, high expression levels of *O*-GlcNAc (*p* = 0.013) or PKM2 (*p* < 0.001) were positively associated with the risk for cancer recurrence.

**Figure 6:**
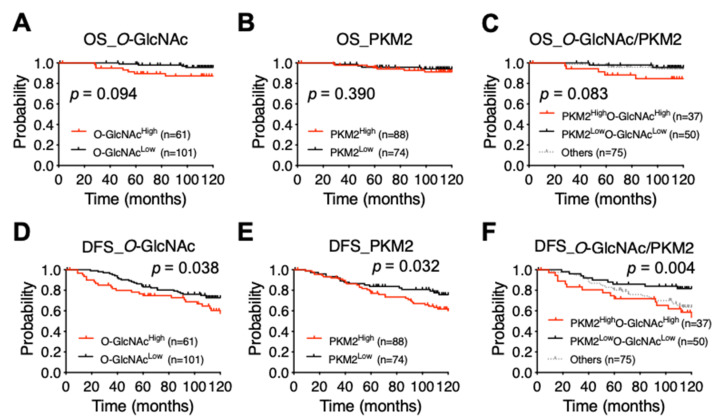
Survival of patients with ER^+^/HER2^−^ luminal BC according to the levels of *O*-GlcNAc and PKM2. (**A**–**C**) The Kaplan–Meier analysis of overall survival (OS) based on the expression level of *O*-GlcNAc (**A**) or PKM2 (**B**) alone or both (**C**). (**D**–**F**) Elevated expression of *O*-GlcNAc (**D**) or PKM2 (**E**) alone or both (**F**) is significantly correlated with 10-year DFS. For group comparisons, *p*-values for a single-marker (high vs. low) or double-marker (*O*-GlcNAChighPKM2high vs. *O*-GlcNAClowPKM2low) statuses were determined by log-rank test.

Furthermore, samples were stratified into two groups using the mean Q score as a threshold. A Kaplan–Meier survival analysis revealed that high expression of *O*-GlcNAc (*O*-GlcNAcHigh, *p* = 0.094), PKM2 (PKM2High, *p* = 0.390), or the combined double-marker status (*O*-GlcNAcHighPKM2High vs. *O*-GlcNAcLowPKM2Low, *p* = 0.083) were associated with worse overall survival, supporting a trend toward statistical significance (Figure 6A–C). Interestingly, high expression levels of *O*-GlcNAc (*O*-GlcNAcHigh, *p* = 0.038) or PKM2 (PKM2High, *p* = 0.032) were significantly associated with worse DFS (Figure 6D–F). Importantly, the combined double-marker status offered an even greater statistical significance (*O*-GlcNAcHighPKM2High vs. *O*-GlcNAcLowPKM2Low, *p* = 0.004) (Figure 6F). These results suggest that high expression levels of *O*-GlcNAc and PKM2 are associated with a high risk of recurrence and worse DFS.

### 3.3. Logistic Regression and Receiver Operating Characteristic (ROC) Curve Analyses

We next sought to evaluate the impact of PKM2 and *O*-GlcNAc combined with a clinical parameter (invasive tumor size, T stage, or CTS5 that integrates four clinical properties [12]) on the risk of 10-year DFS using multivariate Cox regression analysis. As shown in Table S5, the invasive tumor size-*O*-GlcNAc-PKM2 Cox analysis revealed that invasive tumor size and *O*-GlcNAc served as significant factors, whereas PKM2 was a virtually significant element. Similar results were also found for the T stage-*O*-GlcNAc-PKM2 analysis (Table S6). For the CTS5-*O*-GlcNAc-PKM2 multivariate Cox analysis, *O*-GlcNAc was significantly associated with DFS (Table S7), indicating that *O*-GlcNAc is a good prognostic marker in this multivariate Cox analysis. CTS5 also had a trend to be statistically significant. PKM2 expression nonetheless had no significance. It was likely that there was less information that could be provided by PKM2 in this multivariate analysis, perhaps because of the overlapped signals from *O*-GlcNAcylated PKM2, particularly in the *O*-GlcNAChighPKM2high cases (Figure 4).

**Figure 7:**
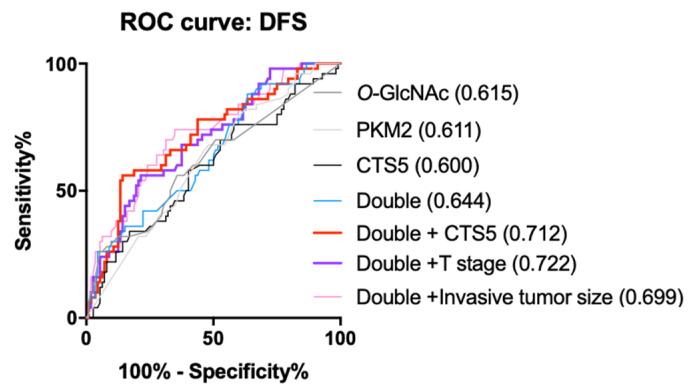
ROC curve analysis in HR^+^/HER2^−^ luminal BC. ROC curve analyses using the *O*-GlcNAc (dark grey line), PKM2 (light grey line), CTS5 (black line), PKM2–*O*-GlcNAc (Double, blue line) the Double–CTS5 (red line) models, the Double–T stage (purple line), and the Double–Invasive tumor size (pink line). Values in parentheses refer to the area under curve (AUC).

Next, we built the logistic regression models using PKM2, *O*-GlcNAc, and/or a clinical parameter (invasive tumor size, T stage, or CTS5) to evaluate the risk of the 10-year DFS outcome. Tables S8–S10 show that each marker (PKM2, *O*-GlcNAc, and an individual clinical parameter (invasive tumor size, T stage, or CTS5)) significantly contributed to the DFS outcome per triple-marker model. Since ROC analysis provides a means to assess the diagnostic ability of a binary classifier system and select possibly optimal models [50], we next conduct ROC analysis to comparing the performance of PKM2 and *O*-GlcNAc in combination with another clinical parameter (invasive tumor size, T stage, or CTS5). Interestingly, the ROC curve analyses revealed that the best performance was seen for the *O*-GlcNAc-PKM2-T stage model (AUC = 0.722), as compared with the *O*-GlcNAc-PKM2-the invasive tumor size model (AUC = 0.699) and the CTS5-*O*-GlcNAc-PKM2 model (AUC = 0.712) (Figure 7). A reduced performance was seen for a single-marker alone (PKM2, *O*-GlcNAc, or CTS5) or the *O*-GlcNAc-PKM2 model (Figure 7). Together, these results suggested that the expression of *O*-GlcNAc and PKM2 provided extra information in addition to the clinical factors. Importantly, T stage, PKM2, and *O*-GlcNAc served as the best prognostic discriminators in the 10-year DFS outcome, offering an improved prognostic indicator for HR^+^/HER2^−^ luminal BC.

## 4. Discussion

Although the expression profiling of risk genes across BC genomes has provided useful information of high genomic risk for HR^+^/HER2^−^ BC [51], the dysregulated metabolic landscape engaged in BC progression and prognosis remains largely unclear. In this study, analysis of large databases revealed that high expression of PKM2 was significantly associated with worse overall survival in luminal tumors. We thus evaluated the expression of PKM2 (a key Warburg-metabolism protein) and *O*-GlcNAc (a nutrient-status integrator) in a retrospective cohort of luminal HR^+^/HER2^−^ BC. Interestingly, high expression of *O*-GlcNAc (*p* = 0.038) or that of PKM2 (*p* = 0.032) was significantly associated with poor 10-year DFS. Remarkably, the combination of the two markers had an even greater significance (*p* = 0.004). The logistic regression analysis also supported this conclusion. Together, the high clinical relevance of the high expression of PKM2 or *O*-GlcNAc with poor 10-year DFS in this retrospective study agrees well with the findings from survival analysis of the METABRIC dataset, as well as other reports regarding their contribution to more aggressive BC behavior [30,42,43,52]. Importantly, these results suggest that they might serve as potential new prognostic markers in luminal HR^+^/HER2^−^ BC.

Clinicopathological factors, particularly TN, have been commonly used in the decision-making process for selecting adjuvant treatments for ER^+^/HER2^−^ BC [15]. Despite a good prognostic value for those clinical parameters, the overall concordance among different parameters in each patient is often heterogeneous. In a meta-analysis of ER^+^ BC patients receiving 5-year endocrine therapy, the risk of late recurrence is found to range from 10% to 41% for up to 20 years, although there is a strong correlation of distant recurrence with the original TN status [8]. Current molecular diagnostics also provides predictive and prognostic values [15,16,17,18,20,21,22]. Despite this, there have been limitations in predicting the risk of recurrence and treatment resistance, particularly low aggressive ER^+^/HER2^−^ tumors [53].

In the present study, we added the expression of PKM2 and *O*-GlcNAc as two potential biomarkers because other groups and we have found their potential relevance in breast cancers [30,31,41,42,43,46]. We have employed IHC analysis and found that levels of PKM2 and *O*-GlcNAc were highly associated with the disease progression and survival of breast cancer patients. We have repeatedly conducted an immunohistochemical analysis of PKM2 and *O*-GlcNAc levels in breast cancer samples and found our results were consistent and reproducible. We have also conducted multi-levels of experiments in validating our findings, including gel filtration, western blot analysis, and IHC of tissue blocks to confirm our antibody tool is reliable. Compared to molecular diagnostics using mRNA- and DNA-based markers, we consider our markers to have great advantages in clinical practice because of the simple and cost-effective approach using IHC has also been widely used in routine diagnoses, such as HER2 staining, offering a great advantage in routine clinical practice.

Maximum likelihood estimation (MLE) provides a robust approach to determine the optimal cutoff for Cox model analysis. The key property of an MLE is a normal-distribution sampling when (1) the parameter, along with all other parameters in the model, is not at a boundary, and (2) sample size is usually assumed large in order to converge [54]. The two parameters (PKM2 and *O*-GlcNAc) in this cohort study were not normally distributed (the *p*-value of the normality test: *O*-GlcNAc, *p* < 0.001; PKM2, *p* < 0.001), possibly because of the small sample size. There were several boundaries in the PKM2 data (Q = 300, *n* = 40), which limited the subsequent Cox regression and logistic regression analysis. Therefore, no cutoff values were used in performing Cox regression and logistic regression analysis.

Notably, *O*-GlcNAc, PKM2, invasive tumor size, or T stage was significantly associated with the DFS outcome in univariate Cox analyses (Table S4). No significance was found for the N stage. Nodal status has less impact in the luminal A subtype, in which the tumor biology is less aggressive. This has been seen from the inclusion of node-positive patients in commercially available gene expression risk predicting panels in their clinical trials (Oncotype Dx, MammaPrint, Prosigna, etc.) [53]. In clinical practice, luminal A tumors are endocrine-sensitive, and the omission of chemotherapy in node-positive patients is also common. These patients enjoy a substantially better prognosis despite lymph node metastasis. On the other hand, the HR^+^ cases were analyzed in this study. If a tumor is ER-negative, it was recruited because PR was positive. The progesterone pathway downstream of ER is still active despite a negative ER expression. However, the major reason for no impact from ER status is the small number of ER-negative cases (*n* = 4) in this study. We were, however, not able to evaluate the impact of Ki-67 in this investigation because of many missing values (*n* = 83).

Multivariate Cox regression analysis revealed that *O*-GlcNAc and a clinical parameter (invasive tumor size, T stage, or CTS5) had significant impacts on the DFS outcome and that PKM2 was virtually a significant factor in the triple-marker models.

Further logistic regression models and ROC curve analyses, interestingly, revealed that the T stage-*O*-GlcNAc-PKM2 triple model exhibited the best performance (Table S7, AUC = 0.722) when compared with the invasive tumor size-*O*-GlcNAc-PKM2 model (AUC = 0.699) and the CTS5-*O*-GlcNAc-PKM2 model (AUC = 0.712). These results suggested that *O*-GlcNAc, PKM2, and T stage together serve as good prognostic discriminators in ER^+^/HER^−^ patients. It is likely that *O*-GlcNAc and PKM2 provide extra metabolic information in addition to the clinical information provided by the T stage. Given an intrinsically heterogeneous nature in breast tumors, further investigation is needed to substantiate our findings using a large-size cohort.

The complex interplay of prognostic factors and adjuvant treatments might dwindle the pure prognostic value of PKM2 and *O*-GlcNAc, which could have been shown in an untreated population. Despite this, we consider that new biomarkers must be evaluated over contemporary treatment to be clinically useful, which is critical to the rational development of therapeutic treatments. Taken together, the information of the expression of PKM2 and *O*-GlcNAc offers useful complementary information to clinicopathological variables.

Genomic assays providing individual risk estimates have been used to aid in adjuvant treatment decisions, particularly regarding the indication of adjuvant chemotherapy in ER^+^/HER2^−^ BC [15]. These assays comprise proliferation-related genes, which are associated with chemosensitivity, as well as proliferation-independent genes related to immune activity; thus, they are also predictive of chemotherapy benefits in different risk groups [16,19]. Our results demonstrated that the expression of PKM2 and *O*-GlcNAc could provide extra information to clinicopathological features to predict the prognostic outcome of BC patients representing an additional layer of information from metabolism. It is likely that crosstalk exists between metabolism and signaling that affects tumor progression [55,56], microenvironmental change [57], and perhaps endocrine therapy resistance [58,59], which in turn contribute to recurrence. Further investigation is needed to better understand the mechanisms by which *O*-GlcNAcylation and PKM2 contribute to the recurrence of luminal BC.

Our study population was based on a Taiwanese cohort of Chinese Han ethnic background in East Asia, where the frequencies of premenopausal patients and the luminal subtype in young patients are higher than those in the West [60]. It is speculated that there might be a complex interplay among the genetic background and environmental factors under the active ovarian function of young women in this region, which leads to a high premenopausal incidence of BC.

The Asian Breast Cancer Cooperative Group 2019 consensus recommends that the treatment of Asian women with luminal BC, particularly for those at the premenopausal stage, requires special considerations, distinct from international guidelines [61].

The strength of this study is that the clinical outcome data were available for all patients and tissue samples were well-characterized. We followed McGuire’s guidelines to explore *O*-GlcNAc and PKM2 statuses as the new prognostic markers [62]. The *O*-GlcNAc and PKM2 statuses were measured in the same laboratory, with all personnel blinded to clinical outcome data. Our study translates a simple metabolism-based IHC analysis in the context of recurrence to clinical applications. The limitation of this study is that we have a selection of 162 HR^+^/HER2^−^ patients with a minimum follow-up of 10 years available from a single medical center in this retrospective analysis. A definitive study of the double marker status, including patients from multi-clinical centers, will be required to provide a solid foundation in a different cohort of patients. The need to further validate the prognostic value of the double-marker status to predict the risks of recurrence in a different cohort study population is important to ascertain this metabolism-based assy.

In summary, *O*-GlcNAc and PKM2 served as potentially independent prognostic markers in HR^+^/HER2^−^ luminal BC in this pilot study. The combination of *O*-GlcNAc-PKM2 with CTS5 provides excellent prognostic accuracy. These results highlight the potential benefits of a metabolism-based risk assay to improve decision-making regarding adjuvant treatment options.

## Figures and Tables

**Table 1:** Clinical properties of patients.

Factors		Non-Recurrent	Recurrent	*p*-Value
		No. (%), *n* = 112	No. (%), *n* = 50	
Molecular subtype	Luminal A	73 (65.2)	35 (70.0)	0.548
	Luminal B	39 (34.8)	15 (30.0)	
Age (years)	Median (IQR)	51.0 (16.0)	53.0 (22.0)	0.517
Age (years)	≤ 50	51 (45.5)	20 (40.0)	0.512
	> 50	61 (54.5)	30 (60.0)	
Diabetes mellitus	No	104 (92.9)	45 (90.0)	0.542
	Yes	8 (7.1)	5 (10.0)	
Operation type	Mastectomy	62 (55.4)	27 (54.0)	0.873
	Breast	50 (44.6)	23 (46.0)	
	conservation			
Invasive tumor size (cm)	Median (IQR)	1.9 (1.4)	2.4 (1.7)	0.019
SBR grade	1	13 (11.6)	5 (10.0)	0.806
	2	61 (54.5)	30 (60.0)	
	3	38 (33.9)	15 (30.0)	
Estrogen receptor	Negative	4 (3.6)	0	0.312
	Positive	108 (96.4)	50 (100.0)	
Progesterone receptor	Negative	11 (9.8)	3 (6.0)	0.553
	Positive	101 (90.2)	47 (94.0)	
Ki67 index (%)	< 25%	30 (26.8)	17 (34.0)	0.006
	≥ 25%	16 (14.3)	16 (32.0)	
	Missing	66 (58.9)	17 (34.0)	
T stage	T1a	0	1 (2.0)	0.014
	T1b	8 (7.1)	1 (2.0)	
	T1c	54 (48.2)	14 (28.0)	
	T2	45 (40.2)	30 (60.0)	
	T3	5 (4.5)	3 (6.0)	
	T4	0	1 (2.0)	
N stage	N0	56 (50.0)	21 (42.0)	0.353
	N1	40 (35.7)	22 (44.0)	
	N2	12 (10.7)	3 (6.0)	
	N3	4 (3.6)	4 (8.0)	
Stage	I	40 (35.7)	9 (18.0)	0.067
	II	55 (49.1)	33 (66.0)	
	III	17 (15.2)	8 (16.0)	
Chemotherapy	No	19 (17.0)	7 (14.0)	0.635
	Yes	93 (83.0)	43 (86.0)	
Hormone therapy	No	3 (2.7)	2 (4.0)	0.645
	Yes	109 (97.3)	48 (96.0)	
Radiotherapy	No	57 (50.9)	23 (46.0)	0.565
	Yes	55 (49.1)	27 (54.0)	

## Data Availability

The information generated and analyzed in the current study is available from cBiopotal (https://www.cbioportal.org (accessed on 25 March 2021)).

## Supplementary Materials

The following are available online at https://www.mdpi.com/article/10.3390/diagnostics11081460/s1, Figure S1: Survival analysis of PKM2 basal subtype and HER2-enrich subtype BC., Table S1: Spearman’s correlation analyses between *O*-GlcNAc and each of parameters, Table S2: Spearman’s correlation analyses between PKM2 and each of parameters, Table S3: Spearman’s correlation analyses between CTS5 and each of parameters, Table S4: Univariate Cox regression analysis to DFS, Table S5: Mutivariate Cox regression analysis of invasive tumor size, *O*-GlcNAc, and PKM2 for DFS, Table S6: Mutivariate Cox regression analysis of T stage, *O*-GlcNAc, and PKM2 for DFS, Table S7: Mutivariate Cox regression analysis of CTS5, *O*-GlcNA, and PKM2 for DFS, Table S8: Logistic regression analysis of CTS5, O-GlcNAc, and PKM2 for DFS, Table S9: Logistic regression analysis of invasive tumor size, *O*-GlcNAc, and PKM2 for DFS, Table S10: Logistic regression analysis of T stage, *O*-GlcNAc and PKM2 for DFS.
