# Supplementary material for: Prognostic Significance of O-GlcNAc and PKM2 in Hormone Receptor-Positive and HER2-Nonenriched Breast Cancer
Source: Diagnostics (Basel). 2021 Aug 12;11(8):1460. doi: 10.3390/diagnostics11081460 (PMC8392504; doi:10.3390/diagnostics11081460)
Supplement: Supplementary file 1 [file diagnostics-11-01460-s001.zip › diagnostics-1282274-supplementary.pdf]

diagnostics

Article

Prognostic Significance of *O*-GlcNAc and PKM2 in Hormone Receptor-Positive and HER2-Nonenriched Breast Cancer

This PDF file includes:

Figures S1

Tables S1 to S10

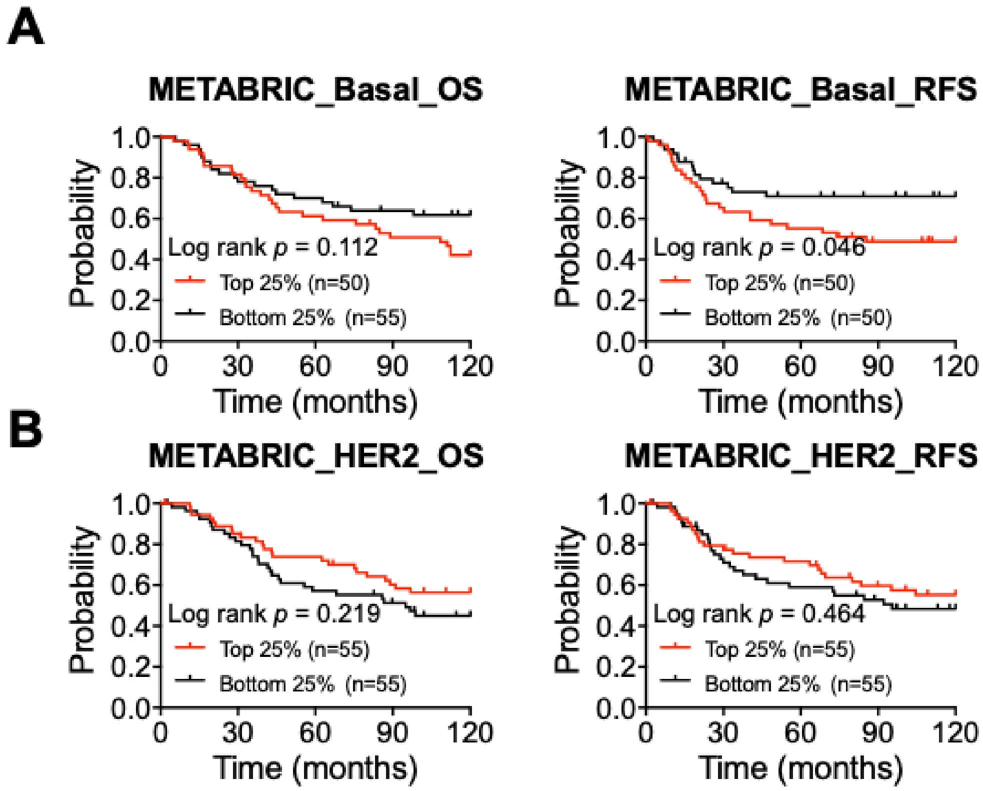

Figure S1. Survival analysis of PKM2 basal subtype and HER2-enrich subtype BC. (A–B) Expression of PKM2 is not significantly correlated with 10-year OS and RFS in both basal subtype (A) and HER2-enriched subtype BC. For group comparisons, *p* values were determined by log-rank test.

Table S1. Spearman's correlation analyses between O-GlcNAc and each of parameters.

Parameters	Correlation analysis - *O*-GlcNAc (Q)
	Correlation Coefficient
Luminal Type	0.143
Age	−0.023
Diabetes mellitus	0.043
Invasive tumor size	0.019
SBR Grade	0.108
Estrogen receptor (01)	0.063
Estrogen receptor (%)	−0.021
Progesterone receptor (01)	0.109
Progesterone receptor (%)	0.087
Ki67 index	83 missing values
T stage	0.055
N stage	−0.119
Stage	−0.079
PKM2 (Q)	0.190
CTS5	−0.014

Table S2. Spearman's correlation analyses between PKM2 and each of parameters.

Parameters	Correlation analysis-PKM2 (Q)
	Correlation Coefficient
Luminal Type	−0.129
Age	0.089
Diabetes mellitus	−0.090
Invasive tumor size	−0.293
SBR Grade	−0.204
Estrogen receptor (01)	−0.021
Estrogen receptor (%)	0.116
Progesterone receptor (01)	0.149
Progesterone receptor (%)	0.107
Ki67 index	83 missing values
T stage	−0.225
N stage	−0.141
Stage	−0.229
O-GlcNAc (Q)	0.190
CTS5	−0.249

Table S3. Spearman's correlation analyses between CTS5 and each of parameters.

Parameters	Correlation analysis-CTS5
	**Correlation Coefficient**
Luminal Type	0.409
Age	0.172
Diabetes mellitus	0.079
Invasive tumor size	0.747
SBR Grade	0.499
Estrogen receptor (01)	0.043
Estrogen receptor (%)	−0.122
Progesterone receptor (01)	−0.141
Progesterone receptor (%)	−0.318
Ki67 index	83 missing values
T stage	0.679
N stage	0.736
Stage	0.792
O-GlcNAc (Q)	−0.014
PKM2 (Q)	−0.249

Table S4. Univariate Cox regression analysis to DFS.

Parameters	Univariate analysis		
	**Coefficient**	**HR (95% CI)**	***p*-value**
Luminal Type (A *vs.* B)	−0.119	1.126 (0.615–2.064)	0.700
Age	0.007	1.007 (0.982–1.033)	0.572
Diabetes mellitus	−0.383	0.682 (0.270–1.718)	0.416
Invasive tumor size	0.215	1.239 (1.035–1.485)	0.020
SBR Grade	−0.051	0.950 (0.603–1.498)	0.232
Estrogen receptor (%)	<0.001	1.000 (0.991–1.009)	0.995
Progesterone receptor (%)	−0.002	0.998 (0.990–1.007)	0.697
Ki67 index (%)		83 missing values	
T stage	0.655	1.925 (1.238–2.991)	0.004
N stage	0.096	1.101 (0.804–1.507)	0.549
Stage	0.286	1.331 (0.888–1.995)	0.166
O-GlcNAc	0.007	1.007 (1.004–1.011)	<0.001
PKM2	0.003	1.003 (1.000–1.006)	0.048
CTS5	0.258	1.294 (0.944–1.774)	0.109

Table S5. Mutivariate Cox regression analysis of invasive tumor size, *O*-GlcNAc, and PKM2 for DFS.

Parameters	Multivariate analysis		
	**Coefficient**	**HR (95% CI)**	***p*-value**
Invasive tumor size	0.293	1.340 (1.106–1.623)	0.003
O-GlcNAc	0.006	1.006 (1.002–1.010)	0.002
PKM2	0.003	1.003 (1.000–1.007)	0.061

Table S6. Mutivariate Cox regression analysis of T stage, *O*-GlcNAc, and PKM2 for DFS.

**Parameters**	**Multivariate analysis**		
	**Coefficient**	**HR (95% CI)**	***p*-value**
T stage	0.785	2.193 (1.376–3.495)	<0.001
O-GlcNAc	0.006	1.006 (1.002–1.009)	0.003
PKM2	0.003	1.002 (1.000–1.007)	0.055

Table S7. Mutivariate Cox regression analysis of CTS5, *O*-GlcNA, and PKM2 for DFS.

**Parameters**	**Multivariate analysis**		
	**Coefficient**	**HR (95% CI)**	***p*-value**
CTS5	0.308	1.360 (0.986–1.877)	0.061
O-GlcNAc	0.006	1.006 (1.002–1.010)	0.003
PKM2	0.003	1.003 (0.999–1.006)	0.125

Table S8. Logistic regression analysis of CTS5, *O*-GlcNAc, and PKM2 for DFS.

	**Coefficient**	**SE**	***p*-value**
CTS5	0.525	0.215	0.018
O-GlcNAc (Q)	0.007	0.003	0.018
PKM2 (Q)	0.005	0.002	0.028
Constant	−3.883	1.002	<0.001

Table S9. Logistic regression analysis of invasive tumor size, *O*-GlcNAc, and PKM2 for DFS.

	**Coefficient**	**SE**	***p*-value**
Invasive tumor size	0.356	0.135	0.008
O-GlcNAc (Q)	0.007	0.003	0.025
PKM2 (Q)	0.005	0.002	0.018
Constant	−2.968	0.663	<0.001

Table S10. Logistic regression analysis of T stage, *O*-GlcNAc and PKM2 for DFS.

	**Coefficient**	**SE**	***p*-value**
T stage	1.006	0.316	0.001
O-GlcNAc (Q)	0.007	0.003	0.032
PKM2 (Q)	0.005	0.002	0.014
Constant	−3.757	0.808	<0.001
